# Restoring Somatotopic Sensory Feedback in Lower Limb Amputees through Noninvasive Nerve Stimulation

**DOI:** 10.34133/cbsystems.0243

**Published:** 2025-04-29

**Authors:** Andrea Demofonti, Marco Germanotta, Andrea Zingaro, Gaia Bailo, Sabina Insalaco, Francesca Cordella, Irene Giovanna Aprile, Loredana Zollo

**Affiliations:** ^1^ Research Unit of Advanced Robotics and Human-Centred Technologies (CREO Lab), Università Campus Bio-Medico di Roma, 00121 Rome, Italy.; ^2^ IRCCS Fondazione Don Carlo Gnocchi ONLUS, 50143 Florence, Italy.

## Abstract

Patients with lower limb amputation experience ambulation disorders since they rely exclusively on visual information in addition to the tactile information they receive from stump–socket interface. The lack of sensory feedback in commercial lower limb prostheses is essential in their abandonment by patients with transtibial amputation (TTA) or transfemoral amputation (TFA). Recent studies have obtained promising results using invasive interfaces with peripheral nervous system presenting drawbacks related to surgery. This paper aims to (a) investigate the potential of transcutaneous electrical nerve stimulation (TENS) as noninvasive means for restoring somatotopic sensory feedback in lower limb amputees and (b) evaluate the effect of the system over a 4-week experimental protocol. The first phase of the study involved 13 participants (6 with TTA and 7 with TFA), and the second one evaluated the long-term effect of TENS on ambulation performance of 2 participants (S1 with TTA and S7 with TFA). The proposed system enhanced participant’s ambulation significantly increasing the body weight distribution between legs (S1: from 134% to 143%, *P* < 0.0055; S7: from 66% to 72%, *P* < 0.0055) and gait symmetry (S1: step length symmetry index from 11% to 5%, *P* < 0.0055; S7: stance phase symmetry index from −4% to −2%, *P* < 0.0055). It led to a postamputation neuropathic pain reduction in S1 (neuropathic pain symptom inventory score diminished from 6 to 0). This demonstrates how TENS enhanced prosthesis embodiment, enabling greater load bearing and more physiological gait patterns. This study highlights TENS as noninvasive solution for restoring somatotopic sensory feedback, addressing the current limitations and paving the way for further research.

## Introduction

Lower limb amputation is a traumatic event for patient quality of life, and it represents a global burden since its annual incidence in Europe and the United States is 87,000 and 240,000 new cases, respectively [1,2]. Notwithstanding the huge advancement in the design of prosthetic devices in terms of support, propulsion, flexibility, and load relief [3], the current commercial lower limb prostheses do not provide users with sensory feedback [4]. Relying exclusively on vision and tactile information from the stump–socket interface [5], patients with lower limb amputation can be affected by ambulation disorders (e.g., slow walking speed [6], gait asymmetry, and overloading of the intact leg [7]), risk of falls [8], and increased metabolic effort during walking [9]. The lack of sensory feedback from the missing limb negatively affects the perception of the prosthesis by inducing a low embodiment [10] and reducing user confidence. All these factors contribute to an increase in the abandoning rate of lower limb prostheses [11].

To restore sensory feedback in lower limb amputees and solve the aforementioned issues related to amputee gait disorders, several approaches have recently been proposed in the literature [11,12].

Several studies have investigated the use of invasive peripheral neural interfaces [13] to restore somatic sensations in lower limb amputees.

The tibial nerve epineural stimulation through composite flat interface nerve electrodes has allowed the elicitation of repetitive sensations of different qualities projected to the missing foot of 2 patients with transtibial amputation (TTA) [14]. Subsequently, the restored sensory feedback led to an increase in stance time, propulsive force, gait symmetry, and prosthesis embodiment in other 3 transtibial amputees [15]. Similarly, intraneural stimulation of the sciatic nerve was tested, through transversal intraneural multichannel electrodes, in 2 patients with transfemoral amputation (TFA). An improvement in walking speed and a decrease in phantom limb pain (PLP) and psychophysical fatigue during task execution were demonstrated.

This led the patients to walk on uneven terrain and drive a conventional car [16–19]. In addition, the agonist–antagonist myoneural interface (AMI) [20] represents a promising surgical approach consisting of 2 serially connected opposite muscle–tendon units so that the contraction and shortening of 1 muscle can induce the stretching of the other [21]. The AMI has been developed in the residual limb of a patient with TTA, and its performance was compared with a control group of 4 patients with traditional TTA. The AMI improved prosthesis control and showed natural reflexive behaviors during stair ambulation that were absent in the control group [22].

The aforementioned feedback strategies are optimal since they are homologous and somatotopic. Indeed, the evoked sensations are similar to the physiological ones in terms of both quality and location. Whether the prosthesis touches the ground, a sensation of touch is elicited on the patient’s phantom foot sole [11]. Nonetheless, the aforementioned promising results are undercut by the drawbacks related to the need for surgery such as the eventual onset of a fibrotic reaction in the proximity of the electrodes and the weak long-term stability of the implant.

Consequently, different noninvasive methods (mainly vibrotactile [23] and electrotactile [24,25] stimulation) have been investigated in the literature.

In [23], a wearable tactile feedback device that delivers short-lasting vibrations around the waist synchronized to gait events was tested in 3 patients with TFA inducing a gait symmetry improvement. Conversely, in [24,25], 3 pairs of electrodes were located on the anterior, lateral, and posterior sides of the stump for providing foot pressure information. This approach was tested in 3 patients with TFA improving temporal gait symmetry, prosthesis confidence, and increasing stability in response to external perturbations.

Despite the promising obtained results, such approaches are not homologous and somatotopic. Therefore, the evoked sensations are not physiologically similar to the desired one in terms of quality and location. Whether the prosthesis touches the ground, a sensation of vibration is elicited on the patient’s stump rather than one of touch in the phantom foot [11].

From this analysis, it can be inferred that the improvement of walking capabilities in patients with lower limb amputation via sensory feedback restoration has been addressed through the use of either invasive or noninvasive neural interfaces. As for the former, the evoked sensations are homologous and somatotopic, but the patient has to undergo a surgery [13–22]; as for the latter, the proposed approaches do not need an implant, but the elicited sensations do not correspond with the physiological ones in terms of location and quality since they are perceived just locally on the stump [23–25].

Recent studies, however, demonstrated that homologous and somatotopic sensations have more advantages than the others. They can increase the intuitiveness and the discrimination accuracy, can reduce the response time and the cognitive load, and can shorten the training phase allowing for immediate and effortless integration of the feedback within the prosthesis [11,26–28].

Subsequently, the authors have investigated the transcutaneous electrical nerve stimulation (TENS) that is widely adopted in current clinical practice for its analgesic effects in the treatment of neck [29], low-back [30], and PLP [31] pain.

For research purpose, TENS represents an excellent compromise among the previous sensory feedback strategies allowing the elicitation of homologous and somatotopic sensation in a noninvasive way. Indeed, TENS uses superficial electrodes placed on the epidermis to electrically stimulate the underlying nerves. Although TENS is less selective than the intraneural and/or epineural stimulation and it is characterized by several technical difficulties (e.g., the need for a wearable stimulator rather than a bench-top one, the presence of cables connecting the stimulator to the electrodes, and the time variability of the stimulation parameters), recent studies have demonstrated TENS efficacy in restoring sensory feedback in upper limb amputees through the median and/or ulnar nerve stimulation. The tactile-elicited sensations were innocuous (mainly perceived as touch/pressure, vibration, and tingling [32–37]) or painful, similar to those experienced during sharp object manipulation [38]. This approach allowed the objects’ stiffness, shape, and texture recognition [39–43] during grasping with a prosthetic hand, ensuring, therefore, bidirectional communication between the prosthesis and the user [44].

Despite good results obtained in the field of upper limb prosthetics, hitherto TENS application to lower limb has not been deeply investigated. The feasibility of evoking somatotopic tactile sensations in the foot has been exclusively evaluated by electrically stimulating the tibial nerve of 15 healthy participants [45,46] and 5 transtibial amputees [47] using electrodes placed along the calf or near the popliteal fossa, respectively.

Although the results obtained from both healthy participants [45,46] and those with TTA [47] are interesting, a full characterization of the sensations and regions that can be elicited by TENS in the patients’ lower limb is missing. Moreover, despite the fact that TFA and TTA have comparable levels of annual incidence [48], a TENS-based sensory feedback restoration system was tested exclusively in patients with TTA. This could be due to the greater severity of TFA than TTA since it is more proximal, it results in the loss of the knee joint, and, mostly, the sciatic nerve is deeper than the tibial one compromising the possibility of a noninvasive electrical stimulation.

In addition, the elicitation via TENS of tactile sensations in the missing lower limb was obtained exclusively under static conditions where the patients lied in a sitting [45,46] or prone [47] position. It has never been investigated whether TENS was able to make patients perceive a phantom limb during walking; therefore, TENS effects on ambulation performance have never been evaluated. Hitherto, such a challenge has been addressed through the use of either invasive neural interfaces or noninvasive methods with the related advantages and disadvantages in terms of ease of use and the typology of elicited sensations. Therefore, the adoption of TENS represents an excellent compromise, and the experimental validation of its effects could represent a milestone in the lower limb prosthetics and a noteworthy progress beyond the state of art.

For this reason, this study proposed a noninvasive nerve stimulation system based on TENS and aims to (a) investigate the potential of TENS as a noninvasive means for restoring somatotopic sensory feedback in participants with different levels of lower limb amputation (i.e., TTA and TFA) and (b) evaluate the effect of the system over a prolonged experimental period (4 weeks in our study).

**Fig. 1.**
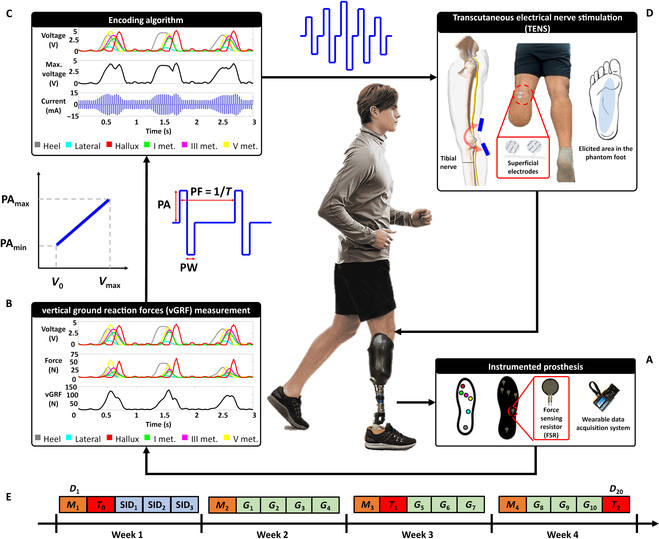
Sensory feedback restoration system overview and study design. (A) The participant with TTA wore his own aesthetic prosthesis with an instrumented insole placed under the foot. (B) Voltage and force signals of the 6 FSRs embedded in the insole and the measured vGRF. (C) The force information was encoded in stimulation patterns (PA, PF, and PW) via a linear modulation. (D) The stimuli were delivered to the participant’s tibial nerve through superficial electrodes placed near the popliteal fossa for the elicitation of referred tactile sensations in the phantom. For the participant with TFA, the working principle of the instrumented prosthesis is the same except for the stimulated nerve (i.e., sciatic rather than the tibial one) and the electrode positioning (i.e., along the femur axis rather than near the popliteal fossa). (E) Overview of the 4-week experimental protocol: *M_i_*, mapping session; SID*_i_*, SID session; *G_i_*, session with G-EO System; *T_i_*, gait analysis; *D_i_*, day.

The first phase of the study aimed to investigate the potential of the proposed system for restoring somatotopic feedback in 13 participants with lower limb amputation divided into 2 groups according to the amputation level. The performed analysis demonstrated the good potentiality of TENS for the elicitation of well-defined, almost natural, and painless tactile sensations in the missing limb of the participants. Relying on the obtained promising results, an instrumented insole was ad hoc developed and embedded in the participant’s aesthetic prosthesis (see Fig. 1A) so that the force values recorded during walking provide the vertical ground reaction force (vGRF) (see Fig. 1B). Force information was linearly translated into electrical stimuli (see Fig. 1C) provided through superficial electrodes to the residual tibial and sciatic nerves of the participant with TTA and TFA, respectively (see Fig. 1D). Subsequently, the second phase of the study aimed to evaluate the effect of TENS on ambulation performance and neuropathic pain of 2 participants (1 with TTA and 1 with TFA) over a 4-week experimental protocol (see Fig. 1E).

## Materials and Methods

### Participants’ recruitment

Patients admitted for rehabilitation at the Fondazione Don Carlo Gnocchi in Rome were consecutively enrolled. The inclusion criteria were (a) age between 18 and 80 years, (b) unilateral TTA or TFA at least 2 months prior to the study, (c) stable clinical conditions, (d) skin integrity, and (e) absence of cognitive deficits. The exclusion criteria were (a) bilateral amputation, (b) open wounds or sores on the residual limb, (c) cognitive deficits, (d) pregnancy, (e) presence of implanted medical device (e.g., cardiac defibrillators, pacemakers, or infusion pumps), and (f) refusal to sign the informed consent.

The study protocol was approved by the Ethics Committee Lazio 1 (protocol no. 1575/CE Lazio 1, 31-12-2020, clinical trial identifier: NCT06160336) in accordance with the Helsinki Declaration and its following amendments; the main aspects of the study were explained to the participants in a comprehensive language, and they signed an informed consent form. All the participants had no previous experience with TENS.

Demographic and clinical characteristics of the enrolled participants are reported in Table S1. Thirteen participants (8 men and 5 women) with a mean age of 61.46 ± 11.12 years were included: 6 of them had TTA, while the other 7 had TFA. The average number of months since the amputation was 18.46 ± 21.46, whereas dysvascular disease (77%) and trauma (23%) were the causes of the amputation. Ten participants (all the patient with TTA and 4 of 7 with TFA) wore aesthetic prostheses, while the remaining 3 had prostheses with mobile knee: in addition, 5 participants can walk without any assistance, whereas 7 used a walker.

As already stated in Introduction, this study evaluated the effect of the proposed sensory feedback restoration system based on TENS on the ambulation and neuropathic pain of 2 participants with different levels of lower limb amputation (S1 and S7) over a 4-week experimental protocol (see Fig. 1E).

The first participant, S1, was an 80-year-old female diagnosed with dysvascular disease. She underwent a TTA of her left leg 4 months prior to joining the research. In addition, she had hypertension and osteoporosis. The second participant, S7, was a 38-year-old male who had a TFA of his left leg due to dysvascular disease occurring 5 months before the study. His condition was complicated by osteomyelitis (caused by *Klebsiella pneumoniae*). He also suffered from diabetes type 1 and chronic renal insufficiency. Both participants adopted aesthetic prostheses and a walker for mobility assistance.

### Study design

This study proposes a noninvasive nerve stimulation system based on TENS and in-depth investigate whether somatotopic sensory feedback in lower limb amputees can be restored.

In the first phase of the study, a full characterization of the regions and sensations that can be electrically elicited through a mapping protocol on the intact and the amputated limbs of 13 participants with amputation (6 with TTA and 7 with TFA) was performed.

The participant was asked to lie down in a prone position on a sterilized medical bed to find the optimal electrode positioning for the nerve of interest stimulation. As for the participants with TTA belonging to group 1, the tibial nerve was stimulated since it is responsible for sensation along the foot sole. Indeed, it originates from the sciatic nerve in the proximity of the poplitea fossa, it runs along the back of the leg, and, near the laciniate ligament, it forks in the median and lateral plantar nerves that afferent to the foot and toes. Conversely, in patients with TFA belonging to group 2, the sciatic nerve was stimulated rather than the tibial one because the access to the latter is not possible because of the level of amputation.

On the basis of biological landmarks, the skin surface above the nerve of interest was located and cleaned using alcohol pad. Then, a neurologist identified the nerve course using electroneurography and guided the location of the stimulation electrodes. Two circular (25 mm in diameter) commercial, autoadhesive, and superficial electrodes (TensCare Ltd., Epsom, UK) were adopted, as they are widely used in the literature [24,25,36,37,45,46,49] because they were developed for electrostimulation on humans; therefore, they are safe and minimize the risk of onset of undesired skin reactions. The electrodes were placed near the popliteal fossa or along the femur axis for the tibial or sciatic nerve stimulation of participants of group 1 or 2, respectively. These locations were selected as they allowed for maximum superficial access to both nerves. Subsequently, both active and counter electrodes were connected to the electric stimulator STG4008 (Multichannel System MCS GmbH, Reutlingen, DE) for TENS application.

In agreement with the literature [32–37,39–47], a symmetric biphasic square wave was used since it induces charge transfer according to phase preventing galvanic process that could cause tissue damages [49–51]. The considered stimulation parameters were the pulse amplitude (PA), the pulse width (PW), and the pulse frequency (PF).

The mapping protocol was composed of 3 phases during which the patient always lies down in a prone position on a sterilized medical bed and during the preliminary phase of identifying the optimal electrode positioning.

1. Perceptual threshold identification. The PW, PF, stimulus duration, and rest between 2 consecutive stimuli were kept constant at 500 μs, 500 Hz, 1 s, and 5 s, respectively. The PA was incremented between 1 stimulus and the subsequent one from 1 mA with a step of 0.1 mA until both the sensory and motor thresholds were reached. The sensory and motor thresholds were defined as the lowest current intensity values needed to evoke a well-defined, referred tactile sensation in the phantom limb and a visible muscle contraction, respectively [52]. Subsequently, 2 different PA values (i.e., *PA*_min_ and *PA*_max_) were identified, respectively, as the minimum and maximum current values able to evoke a well-defined and painless referred tactile sensation in the phantom limb without inducing muscular contraction. The elicited in loco sensation was not recorded since the optimal electrode positioning ensured a negligible local sensation. The total number of stimulus provided to the participants in this phase was variable since perceptual thresholds values were participant-specific.
2. Charge modulation. The PA, PF, stimulus duration, and rest between 2 consecutive stimuli were kept constant at *PA*_max_, 150 Hz, 1 s, and 5 s, respectively. The PW was varied in the range of 100 to 500 μs with a step of 40 μs for a total of 11 stimuli. At the end of the modulation, the PW value at which the participant can feel a well-defined and comfortable sensation was selected.
3. Frequency modulation. The PA, PW, stimulus duration, and rest between 2 consecutive stimuli were kept constant at *PA*_max_, the selected value in the previous phase, 1 s, and 5 s, respectively. The PF was varied in the range of 50 to 500 Hz with a step of 50 Hz for a total of 10 stimuli. The increase in the parameters’ ranges and steps was adopted following the authors’ previous studies [45,46].

For each stimulus, the participant was asked to indicate the location of the evoked sensation adopting pictures representing the missing foot and leg and then to describe it. The descriptors included a 5-point scale for the naturalness (defined as “something that you might encounter in everyday life but not something you became sensitized to due to your condition or its treatment” [53]) ranging from unnatural to natural, a metric of the location of the sensation (i.e., superficial and/or deep), a scale from 0 to 10 for the intensity and pain of the sensation and multiple choices for the quality (touch/pressure, pinch, vibration, tugging, tingling, burning, hot, cold, ankle flexion, ankle extension, toes flexion, toes extension, and nothing). Although the adopted stimulus location and parameters aimed at the elicitation of tactile sensations in the phantom limb, the patients could also choose between qualities other than paresthesia, since the adopted descriptors belong to psychometric questionnaires already used and validated in the literature and cover most of the sensations that can be experienced in daily life [36,37,45,46,53–55].

The mapping protocol was administered to all the participants on both limbs randomly.

Relying on the obtained promising results, the second phase of the study aimed to evaluate the effect of TENS on ambulation performance and neuropathic pain of 2 participants (S1 with TTA and S7 with TFA) over a prolonged period of time.

The 4-week experimental protocol was divided as follows (see Fig. 1E):

• Four mapping sessions (named *M*_1_, *M*_2_, *M*_3_, and *M*_4_) performed at the beginning of each week. The time variability of the proposed sensory feedback system was a crucial factor since, regardless of using the same input parameters, the output could be different over days because of issues such as the electrodes’ displacement and wear, skin sweating, and variation of tissue electrical impedance [56]. Therefore, the aim of these sessions was to manually recalibrate the perceptual thresholds and characterize the elicited sensation’s location and quality on both limbs.
• Three stimulus intensity discrimination (SID) sessions (named SID_1_, SID_2_, and SID_3_) performed in 3 consecutive days of the first week aiming to evaluate the participant’s capability to recognize stimuli with different levels of intensity. Indeed, during the adoption of the proposed sensory feedback restoration system in a realistic scenario, the stimulation current delivered to the participant will assume different intensity levels depending on the load applied to the ground while walking. Consequently, training the patient in recognizing stimuli with different levels of intensity may enable him to discriminate the various gait cycle phases relying exclusively on TENS and, thus, potentially improving ambulation performance. In accordance with the results obtained in *M*_1_, 3 levels of intensity were settled: *PA*_min_, *PA*_max_, and their middle value. The protocol was divided into a familiarization phase in which the levels (low, medium, and high) were presented in order, 3 times each, and then a validation phase in which 45 stimuli (15 for each level) were provided in random way. The participant, lying in a prone position on a sterilized medical bed blindfolded and acoustically shielded, had to recognize each stimulus, discriminate the intensity, and refer it. During the whole session, the PW, PF, stimulus duration, and rest between the stimulus and the following one were kept constant at 500 μs, 500 Hz, 1 s, and 5 s, respectively [46]. The number of intensity levels, their repetitions, and the whole SID experimental protocol were adopted following the authors’ previous studies [46]. The reduced number of SID sessions was conceived to allow the patient to adopt the proposed system in realistic scenarios similar to those described in the following sessions.
• Ten sessions (named *G*_1_... *G*_10_) performed with the end-effector gait rehabilitation robot G-EO System (Reha Technology AG, Olten, CH) aiming to train the participant to walk with the proposed sensory feedback restoration system based on TENS under controlled and repeatable experimental conditions. In each session, the participant was asked to wear the instrumented prosthesis and to walk for 20 min at 30 step/min with a step length of 36 cm, while the robot supported the participant’s body weight (BW). During the walking trials, the electrical stimulation of the residual nerve was triggered and modulated by an instrumented insole embedded in participant’s aesthetic prosthesis (see Fig. 1). In addition, the participant was immersed in a virtual reality environment mimicking a walk in the park, whereas a vertical track bar indicating the task completion percentage was displayed on the left side of the screen.
• Three gait analysis sessions performed at the beginning (*T*_0_), in the middle (*T*_1_), and at the end (*T*_2_) of the experimental protocol, where the participant’s performance during ambulation with and without electrical stimulation and the improvement over time were monitored. Gait analysis was performed with the optoelectronic-marker-based system BTS Smart D-500 (BTS Bioengineering, Milan, IT): 8 cameras were geometrically calibrated and mounted on tripods to capture 22 photoreflective spherical (10 mm in diameter) markers’ motion placed on specific anatomical landmarks of participant’s body according to the Davis protocol [57]. In each session, the participant was asked to perform 10 walking trials (5 with stimulation and 5 without it, in a random order) at a self-selected speed along a straight path of 3.20 m. Since the participant was not able to perform a free overground walking due to the clinical condition, he/she was assisted by a walker in each trial. Although the presence of such an aid could alter both kinematic and kinetic features of ambulation [58], it did not affect the performed analysis since the aid was adopted in each repetition, regardless of the tested condition. Therefore, the only difference between the 2 tested conditions (i.e., with or without the proposed stimulation system) in each session (i.e., *T*_0_, *T*_1_, and *T*_2_) were due exclusively to the restoration of sensory feedback via TENS. At the end of each repetition, the participant was asked to refer to the elicited sensations during walking to evaluate whether they were comparable with those evoked during the mapping sessions. This analysis was carried out since all the previous studies evaluating the effect of somatotopic sensory feedback restoration in lower limb amputees were performed exploiting exclusively invasive interfaces [13] and not TENS. Indeed, TENS was only applied to immobile participants lying in a sitting [45,46] or prone [47] position.

Finally, at the beginning (*T*_0_) and the end of the experimental protocol (*T*_2_), a clinical survey on pain-related symptoms was submitted including the numerical rating scale (NRS) [59] and neuropathic pain symptom inventory (NPSI) [60].

### Instrumented prosthesis for sensory feedback restoration

During the walking trials performed by the selected participants, the electrical stimulation of the residual nerve was triggered and modulated by the vGRF.

To measure the force exerted to the ground during walking, an instrumented insole to be inserted under the participant’s own prosthetic foot was ad hoc developed (see Fig. 1).

The insole was made of a polyurethane foam substrate where 6 force sensing resistors (FSRs) (FSR 402 Short Tail, Interlink Electronics Inc., Westlake Village, CA, USA) were placed in correspondence of the heel, the lateral area of the foot, the I, III, and V metatarsals, and the hallux (Fig. 1). The shape, the number, and the placement of FSRs ensured the acquisition of the maximal force exerted by the participant during foot stance, and they have been defined in accordance with preliminary test and other prototypes available in the literature [16–18,61–65]. The sensors had a resolution of 1 N and a maximum measurable weight of 70 N. The relationship between the force value *F* and the voltage output *V* of each sensor was established with a static characterization as explained in [66] so that the sum of the force values of each sensor allowed the measurement of vGRF exerted during walking.

The acquisition system and conditioning circuit (i.e., a voltage divider for each sensor) were placed inside a 3-dimensional printed (polylactic acid) case attached to the prosthesis’ socket during gait, and it had a sampling frequency of 1 kHz. This solution was chosen to ensure a limited wiring keeping gait as natural as possible avoiding the risk of falls.

The electrical stimuli were provided by means of 2 superficial electrodes placed on epidermis, which were changed at the beginning of each walking trial to maximize adhesion with the skin. In addition, the electrodes were covered by a soft silicone liner worn by each participant. This solution was adopted for 2 main reasons: The former was to avoid small perturbations of electrode positioning and the eventual onset of stump pain; the latter was keeping as constant as possible the distances between the electrodes and the underlying nerve so that the sensations elicited during walking were comparable with those evoked during the mapping sessions. Moreover, the adoption of the liner increased the stability of stump–socket interface.

A linear modulation of the amplitude was adopted to minimize the computational burden of the proposed approach and guarantee a stimulation proportional to the force applied on the ground. Force sensation was elicited by means of a train of pulses with fixed PF (500 Hz) and PW (500 μs). The amplitude linear modulation is described as follows:

$$\begin{array}{c} PA\;\left(V\right)=\\ \left\{\begin{array}{cc} 0 & \text{when}\;V\leq V_{0}\\ \frac{\left(PA_{\max}-PA_{\min}\right)\left(V-V_{0}\right)}{V_{\max}-V_{0}}+PA_{\min} & \text{when}\;V_{0}<V\leq V_{\max}\\ PA_{\max} & \text{when}\;V>V_{\max} \end{array}\right. \end{array}$$ (1)

where *PA*_max_ and *PA*_min_ were the amplitudes that evoked the maximum and minimum sensations, respectively; *V*_0_ and *V*_max_ were the sensors supply range (i.e., 0 and 5 V, respectively), and *V* was the maximum readout of the 6 FSRs embedded in the insole (see Fig. 1). The parameters were settled during the mapping protocol to reduce their uncertainty.

It was demonstrated how such an encoding algorithm is able to elicit sensations that can be easily discriminated by the patient during walking [11,19]. Finally, the adopted neuromodulation was already exploited during sciatic nerve intraneural stimulation of patients with TFA contributing to the improvement of the walking speed and the reduction of metabolic cost and phantom pain [16–18].

### Neuropathic pain evaluation

Postamputation pain was assessed in the participant using the following validated patient-oriented measures: the NRS for pain [59] and the NPSI [60].

The NRS is a unidimensional measure of pain intensity to diagnose and quantify pain in adults, in which a respondent selects a number from 0 (no pain) to 10 (worst pain imaginable) that best reflects the intensity of his/her pain [59].

The NPSI is one of the most extensively used instruments for assessing the intensity of neuropathic pain symptoms. It consists of 10 items corresponding to sensory descriptors that can be grouped into 5 dimensions: spontaneous burning pain (burning), spontaneous pressing pain (pressing), paroxysmal pain (paroxysmal), evoked pain (evoked), and paresthesia/dysesthesia. Each dimension can be scored between 0 and 10. In addition, a final score, equivalent to the total of the 10 descriptors and ranging from 0 to 100, can be calculated [60].

### Data collection and analysis

To collect trial data, 2 different custom-made graphic user interfaces (GUIs) were developed in C# using Visual Studio 2019 (Microsoft, Redmond, WA, USA).

The first GUI was adopted during the mapping sessions to characterize the elicited sensations in the phantom limb and record the stimulation parameters. The obtained results were used to evaluate the naturalness, depth, quality, pain, and intensity of the referred sensation in the missing limb. The elicited in loco sensation was not recorded since the optimal electrode positioning and the adopted experimental protocol ensured a negligible local sensation. The injected charge *Q* was calculated as the product of the PA and PW values (*Q* = PA × PW) [56], while its density was determined as the ratio of *Q* to the adopted electrode surface. The repeatibility of the elicited regions among the participants was quantified through the structural similarity index measure (SSIM) [67]. In addition, the GUI was adopted during the SID sessions to record the stimulus intensity level perceived by the participants. A score of 0 or 1 was assigned depending on whether the participant’s answer was correct, and, therefore, a success rate (SR) was introduced as the percentage of rightly classified stimuli compared to the total.

The second GUI was adopted to collect the 6 FSR readouts of the instrumented insole located under the participant’s aesthetic prosthesis to measure vGRF and modulate the stimulation parameters during walking trials.

During gait analysis, lower limb kinematics and spatiotemporal parameters were recorded through the proprietary software of the optoelectronic-marker-based system, whereas 2 cameras were used for recording videos of the trials in frontal and lateral view. To evaluate symmetry between the intact and the amputated limbs, the symmetry index (SI) [6] was calculated as follows:

$$SI=\frac{X_{\text{prosthetic}}-X_{\text{intact}}}{0.5\times \left(X_{\text{prosthetic}}+X_{\text{intact}}\right)}\times 100\%$$ (2)

where *X* is a generic parameter and *SI* = 0% means complete symmetry between the limbs.

The effect of the 4-week experimental protocol on the participant’ PLP was evaluated through NRS [59] and NPSI [60] administered at the beginning (*T*_0_) and the end (*T*_2_) of the experimental protocol.

### Statistical analysis

The proposed analysis was designed as an initial proof-of-concept study to provide evidence of the benefits afforded to patients with different levels of lower limb amputation by sensory feedback restored via TENS. The proposed stimulation system was evaluated over a 4-week experimental protocol since such a duration was conceived to be sufficient to assess the long-term effect of sensory feedback restored via TENS. Therefore, the number of sessions, the repetitions of the tests performed in each of them, and their durations were determined as follows:

• Mapping and SID sessions were designed following the authors’ previous studies [45,46].
• Sessions with G-EO System were designed according to the robot usage guidelines [68].
• The gait analysis sessions for the evaluation of the participant’s performance during ambulation with and without electrical stimulation and the neuropathic pain evaluation sessions were based on [55]: They were performed at the beginning (*T*_0_), in the middle (*T*_1_), and at the end (*T*_2_) of the experimental protocol.

The number of experiments with *α* = 0.05 guaranteed a statistical power for the 2 participants on average of 98% (Cohen’s *d* = 0.83) for sensation characterization, of 90% (*d* = 0.72) for vGRF peak force values, and of 99% (*d* = 0.97) for gait symmetry analysis.

All data were exported and processed offline in MATLAB (R2020a, MathWorks, Natick, MA, USA). They were reported in box plots where the horizontal line denotes the median value, the lower and upper hinges correspond to the 25th and 75th percentiles, the whiskers extend from the hinge to the most extreme data points (i.e., no more than 1.5× interquartile range), and the + signs indicate the outliers. The Shapiro–Wilk test was adopted for evaluating the normality of data distribution and was non-Gaussian. The Mann–Whitney test was adopted to compare results of the 2 groups of participants since it can be used to compare 2 independent samples. The Wilcoxon signed-rank test was applied to compare results of the same selected participants at different time intervals (e.g., *T*_0_, *T*_1_, and *T*_2_) and under different conditions (e.g., with/without TENS) since it can be used to compare 2 dependent and matched samples. Unless otherwise reported in the figures’ caption and text, the significance level *P* value was 0.05, and Bonferroni correction was executed in case of multiple comparisons among groups of data.

## Results

### Characterization of the elicited sensations in participants

A full characterization of the sensations elicited through TENS was performed on the intact and the amputated limbs of participants with TTA (group 1) and TFA (group 2). The stimuli were provided by means of 2 superficial electrodes located near the popliteal fossa to stimulate the tibial nerve of the participants in group 1. Conversely, the electrodes were located along the femur axis for sciatic nerve stimulation in participants of group 2.

**Fig. 2.**
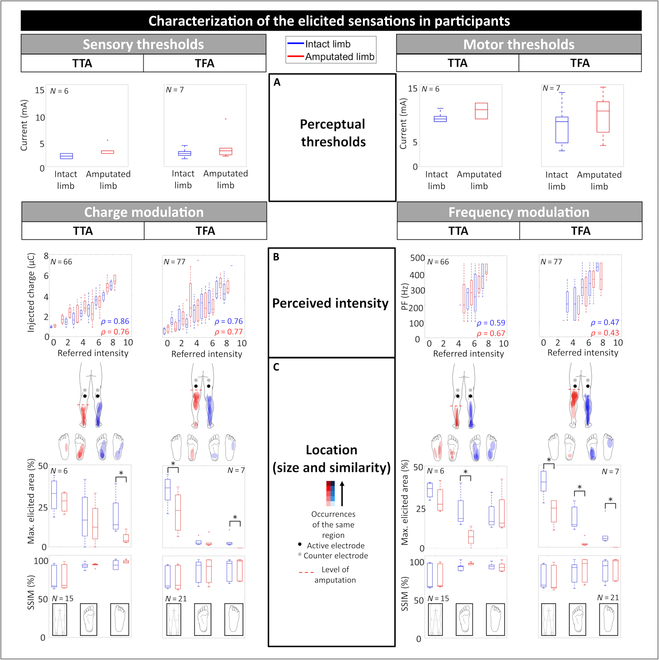
Characterization of the elicited sensations in the participants with TTA and TFA. (A) Perceptual thresholds. (B) Relationships between the stimulus intensity and the intensity referred by the participants. (C) Size and similarity among the participants of the elicited regions at maximum stimulation intensity. The regions depicted with a more vivid color indicate those ones with a higher number of occurrences. Each indicator was evaluated for both intact (blue) and amputated (red) limbs. *P < 0.0125 (Mann–Whitney test with Bonferroni correction).

The sensory and motor thresholds were participant-specific, and they are reported in Fig. 2A and Table S2. In group 1, the sensory thresholds of the intact and the amputated limbs were 1.92 ± 0.49 and 3.08 ± 0.97 mA, respectively, whereas the motor ones were 9.33 ± 0.88 and 11.25 ± 0.84 mA. Similarly, in group 2, the sensory thresholds of the intact and the amputated were 2.57 ± 0.79 and 3.57 ± 2.46 mA, respectively, whereas the motor ones were 8.14 ± 3.44 and 10.21 ± 4.00 mA. Regardless of the level of amputation, the obtained results showed that the perceptual thresholds on the amputated limb were always greater than the intact limb’s ones.

For each stimulus, the participants were asked to indicate the location of the evoked sensation in the missing limb, then to describe it in terms of naturalness (from unnatural to natural), depth (i.e., superficial and/or deep), and quality, and to quantify the referred sensation intensity and pain using a scale from 0 to 10.

Fig. 2B shows the relationship between the stimulus intensity and the referred sensation for the intact and the amputated limbs of participants. As the stimulus increased because of a higher quantity of the injected charge *Q* or the PF, an increase in the referred intensity occurred. Injected charge and referred intensity showed a strong linear correlation quantified through the Pearson coefficient *ρ*: in group 1, *ρ* = 0.86 (*P* < 0.001) and *ρ* = 0.76 (*P* < 0.001) for the intact and the amputated limb, respectively; in group 2, *ρ* = 0.76 (*P* < 0.001) and *ρ* = 0.77 (*P* < 0.001) for the intact and the amputated limb, respectively. Moreover, the stimulation frequency and the referred intensity were moderately correlated: in group 1, *ρ* = 0.59 (*P* < 0.001) and *ρ* = 0.67 (*P* < 0.001) for the intact and the amputated limb, respectively; in group 2, *ρ* = 0.47 (*P* < 0.001) and *ρ* = 0.43 (*P* < 0.001) for the intact and the amputated limb, respectively.

These results were strengthened by the participants’ responses regarding the qualities of the elicited sensations. In both groups, the evoked sensations on the intact limb during charge modulation were mainly described as a natural and painless tingling (58% and 41% for TTA and TFA, respectively), followed by vibration (17% and 30% for TTA and TFA, respectively). For the amputated limb, participant’s description was reversed: Vibration was the most reported quality (56% and 42% for TTA and TFA, respectively), followed by tingling (17% and 30% for TTA and TFA, respectively). Since a similar behavior was experienced in both groups during frequency modulation, it means that an increase in the stimulus provided on the amputated limb leads to an increase in the strength of the sensation. More details regarding the qualities of the elicited sensations in both groups are reported in Table S3.

Fig. 2D shows the elicited regions in each participant in the intact and phantom leg, foot sole, and instep at maximum stimulation intensity during charge and frequency modulation. The areas indicated by the participants were overlapped, and those depicted with a dark color were specified with a higher number of occurrences. As evident from the shades of color, all participants with TTA were able to perceive tactile sensations in different areas of the intact and amputated limbs including regions proximal to electrode placement (e.g., the calf) but especially and most noteworthy in the distal ones (e.g., the foot sole and instep). Conversely, TENS was able to evoke referred tactile sensations in the phantom foot sole of the participants with TFA only in a few rare cases. Moreover, a difference was noted between the limbs in terms of area size, expressed as percentage of figure area shown to the participants for sensation characterization. The obtained results presented good repeatability since the SSIM [67] evaluated among the elicited areas was always greater than 75% [69].

### Modification over time of the elicited sensations

Relying on the obtained promising results, this study evaluated the effects of the proposed system on the ambulation and neuropathic pain of 2 participants with different levels of amputation (S1 with TTA and S7 with TTA) over a 4-week experimental protocol (see Fig. 1E).

**Fig. 3.**
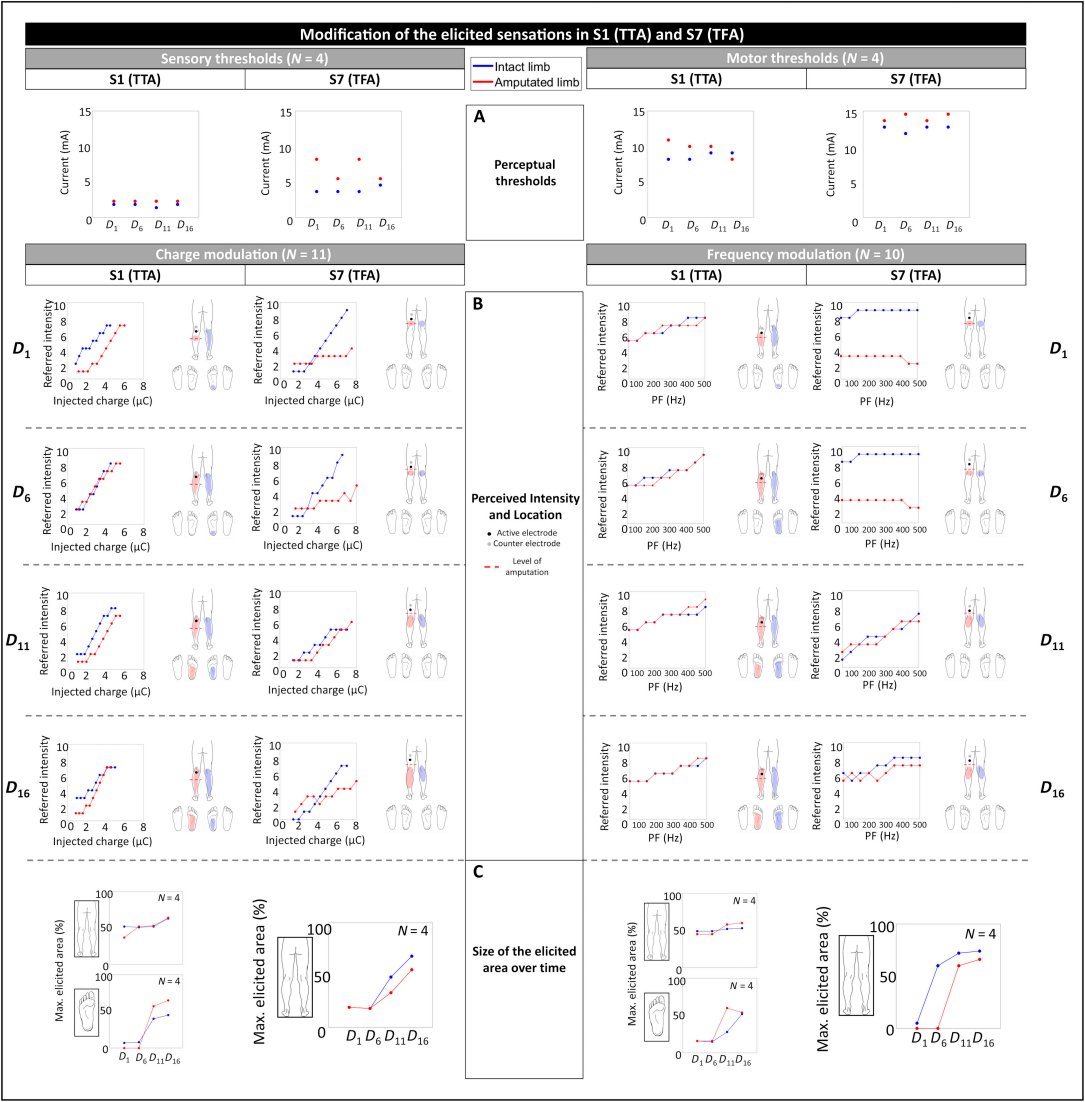
Modification over time of the elicited sensations in the selected participants with TTA (S1) and TFA (S7). (A) Perceptual thresholds. (B) Perceived intensity and location of the elicited sensations. (C) Size of the elicited regions at maximum stimulation intensity quantified through the percentage of covered area with respect to the total available. Each indicator was evaluated for each of the 4 mapping sessions (*D*_1_, *D*_6_, *D*_11_, and *D*_16_) for both intact (blue) and amputated (red) limbs.

Since the time variability of the proposed sensory feedback system was a crucial factor, the mapping session was performed at the beginning of each week (*D*_1_, *D*_6_, *D*_11_, and *D*_16_) to manually recalibrate the perceptual thresholds and characterize the elicited sensation’s location and quality on both limbs. The sensory and motor thresholds for both limbs of the participants in each mapping session are reported in Fig. 3A.

The sensory thresholds of the intact and the amputated limbs of S1 among the sessions were 1.75 ± 0.29 mA [coefficient of variation (CV) = 0.17] and 2.63 ± 0.23 mA (CV = 0.09), respectively, whereas the motor ones were 9.50 ± 0.58 mA (CV = 0.06) and 10.00 ± 0.82 mA (CV = 0.08), respectively. The sensory thresholds of the intact and the amputated limbs of S7 among the sessions were 4.25 ± 0.50 mA (CV = 0.12) and 7.50 ± 1.73 mA (CV = 0.23), respectively, whereas the motor thresholds were 13.75 ± 0.50 mA (CV = 0.04) and 15.50 ± 0.58 mA (CV = 0.06), respectively. The obtained values confirmed the previous results regarding the need to provide a higher amount of charge on the amputated limb than to the intact limb to obtain the same sensation intensity.

Fig. 3B shows the relationship between the stimulus intensity and the referred sensation for the intact and the amputated limbs of the participants among the 4 mapping sessions.

As already encountered in groups 1 and 2, as the stimulus increased because of a higher quantity of injected charge or PF, the 2 selected participants referred an increase in the sensation intensity. The Pearson coefficient *ρ*, evaluated between the injected charge and the referred intensity during each mapping session, showed a significant (*P* < 0.001) strong linear correlation: for S1, *ρ* = 0.98 ± 0.01 for both the intact and the amputated limbs; for S7, *ρ* = 0.98 ± 0.01 and *ρ* = 0.91 ± 0.05 for the intact and the amputated limbs, respectively. A similar trend was noticed in the relationship between the PF and the referred intensity (*P* < 0.001): for S1, *ρ* = 0.96 ± 0.02 for both the intact and the amputated limbs; for S7, *ρ* = 0.82 ± 0.14 and *ρ* = 0.79 ± 0.12 for the intact and the amputated limbs, respectively.

In addition to what has already been described, Fig. 3C shows the sizes of the elicited regions in the intact and phantom leg, foot sole, and instep at maximum stimulation intensity during charge and frequency modulation in each mapping session.

As for the mapping results of S1, they demonstrated how the elicited regions in the amputated limbs expanded over time. In the charge modulation of the first mapping session (first 2 graphs of Fig. 3C starting from the left), TENS was able to elicit natural, painless, and tactile sensations in a localized region (37% of total available area) just below the electrode location and not in the foot sole. Over time, this region expanded along the whole calf to the ankle (54%) and into the foot sole (65%). These results were also confirmed during frequency modulation (fourth and fifth graph of Fig. 3C, starting from the left) where the maximum elicited areas in the phantom leg and foot sole in the last mapping session were 60% and 66%: Indeed, the highest value of PF reached in this phase (500 Hz) was higher than that one (150 Hz) used during charge modulation.

Although the mapping results of S7 were in accordance with those of group 2, the elicited regions in the amputated limb expanded over time likely in S1. In the charge modulation of the first mapping session (third graph of Fig. 3C, starting from the left), TENS evoked natural, painless, and tactile sensations in a localized region (19% of the available area) just below the electrode location, which then expanded along the calf to the ankle (55%). A similar behavior was noticed during frequency modulation where the region increased over time from 16% to 54% (sixth graph of Fig. 3C, starting from the left).

A similar trend was also encountered on the intact limbs of both participants during charge and frequency modulation.

Three SID sessions were performed in 3 consecutive days of the first week (*D*_3_, *D*_4_, and *D*_5_; see Fig. 1A). Exploiting the results obtained in the first mapping session held at *D*_1_, 3 levels of intensity (low, medium, and high) were settled and delivered to the participants in a random way in each session. The participants, blindfolded and acoustically shielded, were asked to recognize stimulus intensity and refer it.

**Fig. 4.**
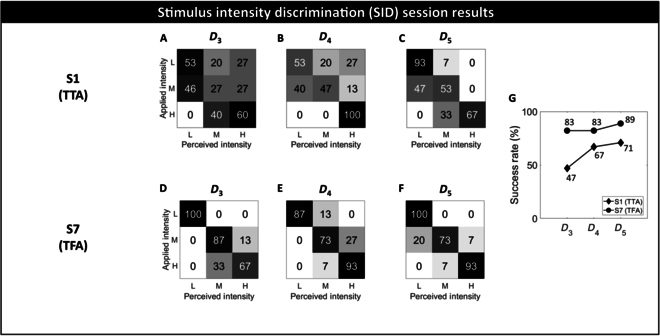
SID sessions results. The participant’s capability in discrimination of 3 different levels of stimulus intensity (low, L; medium, M; high, H) was evaluated through normalized confusion matrices and SR. (A to C and D to F) Normalized confusion matrices of SID_1_, SID_2_, and SID_3_ performed at *D*_3_, *D*_4_, and *D*_5_ of the participants S1 (TTA) and S7 (TFA), respectively. (G) Overall SR value of the participants among sessions.

Fig. 4 shows the normalized confusion matrices and the overall SR values of both participants for the 3 SID sessions.

Both participants showed an improvement in the discrimination of each level of intensity, leading to an increase in the overall SR value from the first session to the last one: from 47% to 71% for S1 and from 83% to 89% for S7.

### Sensory feedback recovery during walking

Relying on the obtained promising results, the second phase of the study aimed to evaluate the effect of TENS on ambulation performance and neuropathic pain of the 2 selected participants over a prolonged period of time.

An instrumented insole was ad hoc developed and embedded in the participant’s aesthetic prosthesis (see Fig. 1A) so that the sum of the force values of each sensor allows the vGRF measurements exerted by the prosthesis during walking (see Fig. 1B). Force information was linearly translated into electrical stimuli (see Fig. 1C) provided through superficial electrodes to the residual tibial and sciatic nerve of S1 and S7, respectively (see Fig. 1D).

**Fig. 5.**
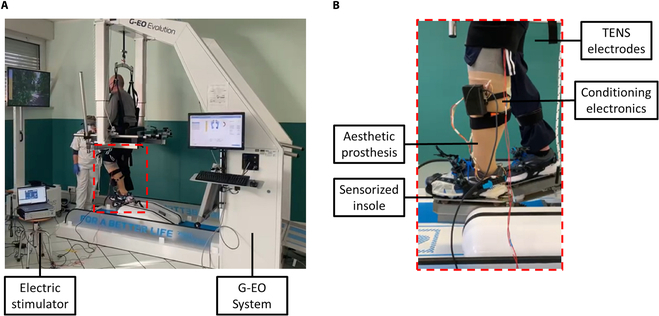
Walking trials with the G-EO system. (A and B) Experimental setup of tests with the end-effector gait rehabilitation robot G-EO System and zoom of the instrumented prosthesis. The electrical stimulation of the residual nerve was modulated by an instrumented insole embedded in the participant’s aesthetic prosthesis and provided through 2 superficial electrodes located near the popliteal fossa for the S1’s tibial nerve stimulation or along the femur axis for the S7’s sciatic nerve stimulation.

The participants adopted the proposed system during 10 sessions (named *G*_1_... *G*_10_) with the end-effector gait rehabilitation robot G-EO System performed in the remaining 3 weeks, aiming to train the participants to walk with the proposed system under controlled and repeatable experimental conditions (see Fig. 5).

Participant performance during ambulation with and without sensory feedback and improvement over time was evaluated through 3 gait analyses performed at the beginning (i.e., *D*_2_, named *T*_0_), in the middle (i.e., *D*_12_, named *T*_1_), and at the end (i.e., *D*_20_, named *T*_2_) of the experimental protocol (see Movie S1).

**Fig. 6.**
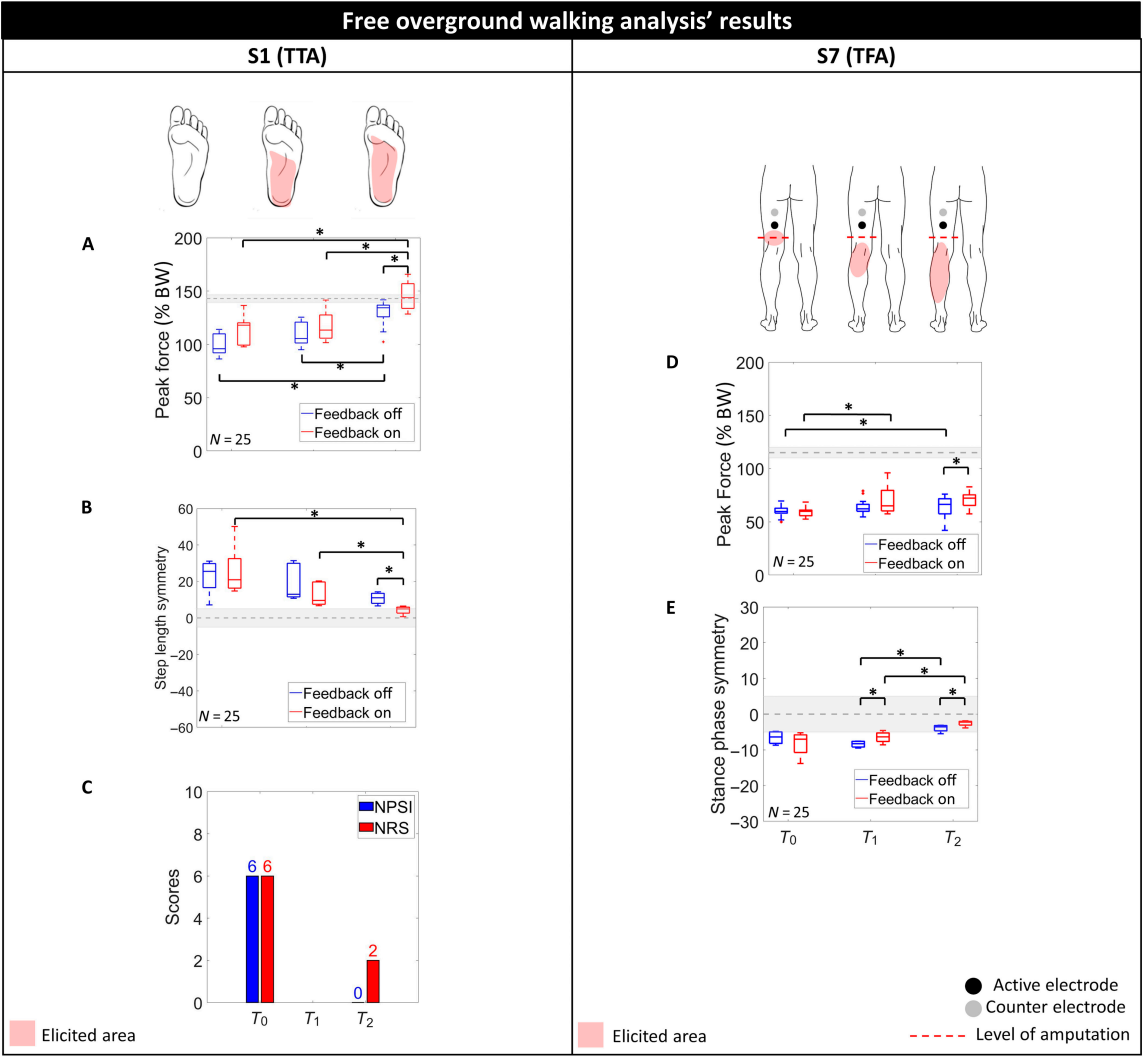
Effect of the proposed TENS-based sensory feedback restoration system on free overground walking and neuropathic pain of the 2 selected participants with TTA (S1) and TFA (S7). (A to C) The elicitation of tactile sensations in the missing foot has significantly increased the peak values of vGRF (expressed as percentage of the participant’s BW) exerted during walking (A) and step length symmetry (B) of S1 and reduced postamputation neuropathic pain quantified in terms of NPSI and NRS scores (C). (D and E) The introduction of sensory feedback has significantly increased the peak values of vGRF exerted during walking (D) and stance phase symmetry (E) of S7. The gray regions (dashed lines with shaded areas) represent the mean values and SDs of age-related physiological values of the relative parameter. *P < 0.0055 (Wilcoxon signed-rank test with Bonferroni correction).

Fig. 6 shows the effects of the proposed system on both participants’ overground walking in terms of spatiotemporal parameters, vGRF, and postamputation neuropathic pain. In addition, the elicited regions in the missing limb during at *D*_1_, *D*_11_, and *D*_16_ are reported.

As far as S1 is concerned, the elicited regions in the amputated limbs expanded over time from a localized region just below the electrode location during the first mapping session to an area covering the 65% of the missing foot sole. The possibility of perceiving the phantom limb during task execution increased the peak values of the vGRF (expressed as percentage of the participants’ BW) exerted by the prosthesis during walking (see Fig. 6A) toward physiological values [70]. At *T*_0_, when the elicited region was located only in the calf and not in the foot, the peak values of vGRF moved from 96% without feedback to 118% with feedback (*P* > 0.0055, Wilcoxon signed-rank test with Bonferroni correction). The same trend was reported at *T*_1_ (from 105% without feedback to 113% with feedback) and mostly at *T*_2_. At the end of the experimental protocol, the participant was able to perceive the missing foot sole, and this led to a significant increase in the peak values of vGRF moving from 134% without TENS to 143% with TENS (*P* = 0.0045 < 0.0055, Wilcoxon signed-rank test with Bonferroni correction). The obtained results confirmed that the introduction of the proposed system significantly improved the participant’s confidence in the prosthesis since a greater load is applied on it.

From a global perspective, the obtained results showed a significant improvement of the participant’s performance from the beginning to the end of the experimental protocol with/without TENS. In absence of sensory feedback, the peak values of vGRF increased from 96% at *T*_0_ to 106% at *T*_1_ and 134% at *T*_2_ ($P_{T_{0}-T_{2}}<0.001$ and $P_{T_{1}-T_{2}}<0.001$). Similarly, in presence of sensory feedback, the peak values of vGRF moved from 118% at *T*_0_ to 113% at *T*_1_ and 143% at *T*_2_ ($P_{T_{0}-T_{2}}<0.001$ and $P_{T_{1}-T_{2}}<0.001$).

The elicitation of tactile sensations in the missing foot was essential also for the improvement of gait symmetry, since the proposed system improved the step length symmetry toward physiological values (*SI* = 0%) in each session (see Fig. 6B). At *T*_0_, step length symmetry moved from 26% without feedback to 21% with feedback. A similar behavior was observed at *T*_1_ when it moved from 16% without feedback to 10% with feedback and mostly at *T*_2_. In the last session, the presence of TENS significantly reduced step length SI from 11% to 5% (*P* = 0.001). Furthermore, the obtained results reported a significant improvement of the participant’s ambulation performance with TENS from the beginning to the end of the experimental protocol: step length SI moved from 21% at *T*_0_ to 10% at *T*_1_ and 5% at *T*_2_ ($P_{T_{0}-T_{2}}<0.001$ and $P_{T_{1}-T_{2}}<0.001$).

As reported in Fig. 3, S7 had never felt any tactile sensation in the missing foot but the elicited region expanded over time from a localized region just below the electrode location during the first mapping session to an area covering the 55% of the missing leg.

Nevertheless, even the possibility of feeling a portion of the missing leg rather than the whole one contributed to an increase in the vGRF peak values exerted on the prosthesis during walking (see Fig. 6D) toward physiological values [70] in each of the 3 sessions. During the first 2 experimental sessions, the elicited regions covered exclusively 19% and 33% of the missing leg, and, therefore, TENS affected in a slight and not significant way: The peak values of vGRF were 58% and 61% without and with feedback at *T*_0_ and 62% and 65% at *T*_1_. At the end of the experimental protocol, the elicited area covered 55% of the missing leg, and this led to a significant improvement of vGRF moving from 66% without TENS to 72% with TENS (*P* = 0.0050 < 0.0055). In addition, the statistical analysis pointed out a significant improvement of the BW distribution between legs between *T*_0_ and *T*_2_ with feedback (*P* < 0.001) and between *T*_0_ and *T*_2_ without it (*P* = 0.0015 < 0.0055).

Similarly to S1, the elicitation of tactile sensations in the missing foot contributed to the gait symmetry since an improvement of stance phase symmetry toward physiological values (SI = 0%) was encountered (see Fig. 6E). At *T*_0_, the impact of TENS on stance phase symmetry was negligible since it was −6% without it and −7% with it. Conversely, the restoration of sensory feedback significantly improved stance phase SI in the remaining 2 sessions: at *T*_1_, SI moved from −8% without feedback to −6% with feedback (*P* = 0.0047 < 0.0055), whereas, at *T*_2_, SI moved from −4% without feedback to −2% with feedback (*P* = 0.0043 < 0.0055).

Furthermore, the obtained results reported the following significant improvement over time of the stance phase SI: It moved from −7% at *T*_1_ to −4% at *T*_2_ without feedback ($P_{T_{1}-T_{2}}=0.0033<0.0055$) and from −6% at *T*_1_ to −2% at *T*_2_ without feedback ($P_{T_{1}-T_{2}}=0.0039<0.0055$).

The obtained results showed that the proposed noninvasive nerve stimulation system based on TENS allowed the participants to feel tactile sensations in all or part of the missing limb during gait and, consequently, to improve BW distribution between legs and gait symmetry toward physiological values.

### Clinical assessment of neuropathic pain

The effect of the 4-week experimental protocol on the participant’ postamputation neuropathic pain was evaluated through NRS [59] and NPSI [60] administered at the beginning (*T*_0_) and the end (*T*_2_) of the study.

As represented in Fig. 6C, on one hand, the participant with TTA (S1) showed a reduction of both pain intensity (NRS score moving from 6 at *T*_0_ to 2 at *T*_2_) and neuropathic pain severity (NPSI score moving from 6 at *T*_0_ to 0 at *T*_2_). On the other hand, the participant with TFA (S7) did not suffer from postamputation neuropathic pain as NRS and NPSI scores were 0 both at *T*_0_ and *T*_2_.

## Discussion

In the first phase of the study, a full characterization of the elicited sensations through TENS was performed on the intact and the amputated limbs of 13 participants with lower limb amputation (6 with TTA and 7 with TFA) divided into 2 groups according to their amputation level.

The obtained results pointed out that the perceptual thresholds of the amputated limb were greater than the intact limb’s ones in both groups (see Fig. 2 and Table S2). This feature could be due to the neural reorganization in the stump following the amputation and the related onset of scar tissue. The increase in skin impedance required providing a higher amount of charge on the amputated limb than on the intact limb, leading to an increase in the strength of the sensation (see Table S3). Indeed, in both groups, the evoked sensations on the intact limb were mainly described as a natural and painless tingling, followed by vibration. Conversely, for the amputated limb, the participant’s description was reversed, and, thus, vibration was the most reported quality, followed by tingling.

The mapping results demonstrated how all the participants with TTA were able to perceive tactile sensations in different distal areas of the intact and amputated limb. Conversely, TENS was able to evoke referred tactile sensations in the phantom foot sole of the participants with TFA only in few rare cases. This may be attributed to anatomical considerations. Indeed, TFA is more severe than TTA as it is more proximal and, most notably, because the sciatic nerve lies deeper than the tibial nerve, limiting the feasibility of noninvasive electrical stimulation.

Such results represented a progress beyond the state of art since, for the first time, a full characterization of the evoked sensations in terms of perceptual thresholds, referred sensation, size, and similarity of the evoked regions was performed in participants with 2 different levels of amputation. This emphasized the adaptability of the proposed approach, as TENS has never been applied to participants with TFA because the only previous study concerning the application of TENS on patients [47] involved only 5 transtibial amputees.

In addition, the experimental setup adopted in [47] consisted of a couple of 10-mm-diameter electrodes placed in the popliteal fossa, a symmetric biphasic square wave with *PW* = 200 μs, *PF* = 150 Hz, PA, and charge density values ranging from 8.5 to 10.5 mA to 27 nC/mm^2^, respectively.

The experimental setup built up in the current authors’ study is similar to that one of [47] with the only variations regarding the electrodes’ diameter (25 mm rather than 10 mm) and the adopted PW (500 μs rather than 200 μs). This ensured the possibility of evoking somatotopic tactile sensations in patients using a strongly smaller range of charge density (ranging from 9 to 14 nC/mm^2^ and from 6 to 15 nC/mm^2^ for participants with TTA and TFA, respectively), minimizing the risk of tissue damages.

Afterward, the long-term effect of TENS was studied on 2 selected participants (S1 with TTA and S7 with TFA), and, therefore, ambulation performance and neuropathic pain reduction were assessed. During walking trials, electrical stimulation was triggered and linearly modulated by the readouts of 6 force sensors of an instrumented insole integrated in the participant’s aesthetic prosthesis.

In the first 2 mapping sessions held at *D*_1_ and *D*_6_, TENS was able to elicit natural, painless, and tactile sensations in a localized region below the electrode location. Nonetheless, over time, this region expanded in such a way that S1 and S7 perceived, respectively, a tingling in the foot sole and in the whole calf in the last 2 sessions held at *D*_11_ and *D*_16_ (see Fig. 3).

Therefore, although both participants had no previous experiences with TENS, the intuitiveness of the proposed approach allowed the patients to perceive referred tactile sensations. This demonstrated that nerves still provided meaningful sensations back to the participants, indicating a residual cortical representation of the phantom lower limb and intact neural pathways. Moreover, this is fostered by a short time since amputation (i.e., 4 and 5 months for S1 and S7, respectively), and the optimal electrode positioning identified through electroneurographic analysis. Although the sensations evoked in the first mapping session were perceived exclusively in reduced regions just below the electrodes, the repetitive and daily use of the proposed TENS-based system emphasized the residual neural pathways and cortical representation of the phantom lower limb, inducing an enlargement of the evoked regions.

As reported in Fig. 6, the restoration of sensory feedback via TENS and therefore the possibility to feel the missing limb led to a significant improvement of both participants’ gait.

As far as S1 is concerned, the introduction of sensory feedback increased the peak values of the vGRF exerted on the prosthesis toward physiological values in each session, mostly in the last session *T*_2_, when the difference between the trials with and without TENS was significant (134% without stimulation to 143% with stimulation, *P* = 0.0045 < 0.0055). This means that the introduction of the proposed system significantly improved the participant’s confidence in the prosthesis since a greater load is applied on it. A similar trend was encountered for step length symmetry since, in the last session *T*_2_, the step length SI significantly reduced from 11% without TENS to 5% with TENS (*P* = 0.0010 < 0.0055). These findings were enhanced by a reduction of postamputation neuropathic pain.

As far as S7 is concerned, although the participant had never felt the foot sole, the possibility of feeling a somatotopic tactile sensation exclusively in the missing calf contributed to the improvement of the ambulation performance in terms of peak values of vGRF and stance phase symmetry. In the first 2 experimental sessions, TENS affected in a slight and not significant way: Conversely, in the last session *T*_2_, the peak values of vGRF moved from 66% without TENS to 72% with TENS (*P* = 0.0050 < 0.0055). Similarly to S1, the elicitation of tactile sensations in the missing foot contributed to gait symmetry since an improvement of stance phase symmetry at *T*_2_ is reported: SI moved from −4% without feedback to −2% with feedback (*P* = 0.0043 < 0.0055).

## Conclusion

This study proposed a noninvasive nerve stimulation system based on TENS and aims to (a) investigate the potential of TENS as a noninvasive means for restoring somatotopic sensory feedback in participants with different levels of lower limb amputation (i.e., TTA and TFA) and (b) evaluate the effect of the system over a prolonged experimental period (4 weeks in our study).

For the first time, a full characterization of the evoked sensations in terms of perceptual thresholds, referred sensation, size, and similarity of the evoked regions was performed in 13 participants with lower limb amputation (6 with TTA and 7 with TFA). In both groups of patients, the proposed system was able to elicit natural, painless, and tactile sensations in the phantom limb with a good level of repeatability (SSIM always greater than 75%). This highlighted the adaptability of the proposed approach, as TENS has never been applied to participants with TFA because the only previous study concerning the application of TENS on patients involved exclusively 5 transtibial amputees.

Subsequently, the effect of TENS on ambulation performance of 2 participants (S1 with TTA and S7 with TFA) was evaluated in terms of lower limb kinematics, spatiotemporal parameters, and vGRF over a 4-week experimental protocol. The possibility for patients to perceive their missing limb again during walking via TENS significantly improved their walking capabilities. The proposed system significantly increased the BW distribution between legs (S1: from 134% to 143%, *P* < 0.0055; S7: from 66% to 72%, *P* < 0.0055) and gait symmetry (S1: step length SI from 11% to 5%, *P* < 0.0055; S7: stance phase SI from −4% to −2%, *P* < 0.0055).

The obtained results represent a huge progress beyond the current state of art and make this study a milestone in the literature of the field. This is the first study demonstrating (a) the potential of a noninvasive sensory feedback restoration system based on TENS for the elicitation of somatotopic tactile sensations in participants with 2 different levels of amputation (TTA or TFA) and (b) the introduction of such a sensory feedback can improve amputee’s ambulation performance and contribute to the reduction of postamputation neuropathic pain. This study paves the way for further investigations since it demonstrated how TENS is able to overcome the main drawbacks of both invasive (i.e, the need for surgery) and noninvasive (i.e., the elicitation of a nonsomatotopic feedback) methods currently adopted in the literature eliciting homologous and somatotopic sensations in the patients’ missing limb in a noninvasive way.

In the future, evidence from a larger population should be gathered to confirm the obtained results. Moreover, the trial should be conducted in a more realistic scenario to evaluate whether unevenness and slopes of the terrain can negatively affect the participant’s ambulation. Subsequently, efforts will be conducted to improve the wearability of the system and increase its chronic use. Algorithms based on machine learning and neural networks will be designed to automatically recalibrate the stimulation parameters avoiding manual operation [56,71–73]. In addition, a miniaturized and wireless solution will be developed for providing the participants with a full wearable system to be used at home in daily life. Finally, to increase the long-term comfort of the system, the currently adopted superficial electrode could be substituted with ultrathin tattoo electrodes easily adaptable to small skin perturbations.

## Data Availability

All data needed to evaluate the conclusions are present in the paper or in the Supplementary Materials. Additional data may be requested from the corresponding author upon reasonable request.
