# Supplementary material for: Restoring Somatotopic Sensory Feedback in Lower Limb Amputees through Noninvasive Nerve Stimulation
Source: Cyborg Bionic Syst. 2025 Apr 29;6:0243. doi: 10.34133/cbsystems.0243 (PMC12038349; doi:10.34133/cbsystems.0243)
Supplement: Supplementary 1 — Tables S1 to S3 Movie S1 [file cbsystems.0243.f1.zip › Revised_Manuscript_with_Supplementary_Materials_Clean_new.pdf]

Supplementary Materials

Table S1. Demographic and clinical characteristic of the enrolled participants

Table S2. Sensory and motor thresholds of the intact and the amputated limbs of the enrolled participants

Table S3. Characteristics of the elicited sensations during charge and frequency modulation of the tibial and sciatic nerve stimulation of the intact and the amputated limbs in the enrolled participants

Movie S1. Sensory feedback restoration system based on TENS: working principle and applications on lower limb amputees

Table 1: Demographic and clinical characteristic of the enrolled participants

ID	Gender	Age [years]	Mass [kg]	Height [cm]	Months since Amputation	Amputated Limb	Etiology	Prosthesis	Walking Aid
**Group 1: TTA**									
**S1**	F	80	56	166	4	L	Dysvascular disease	Aesthetic	Walker
**S2**	M	56	88	174	3	L	Traumatic	Aesthetic	Walker
**S3**	M	56	85	180	10	R	Dysvascular disease	Aesthetic	-
**S4**	M	72	95	172	7	R	Dysvascular disease	Aesthetic	Walker
**S5**	M	66	80	174	10	L	Dysvascular disease	Aesthetic	-
**S6**	F	65	84	156	23	L	Dysvascular disease	Aesthetic	-
**Group 2: TFA**									
**S7**	M	38	68	175	5	L	Dysvascular disease	Mobile knee	Walker
**S8**	M	54	88	180	69	R	Traumatic	Mobile knee	-
**S9**	F	72	49	160	6	R	Dysvascular disease	Aesthetic	Walker
**S10**	F	58	110	173	35	L	Dysvascular disease	Aesthetic	Walker
**S11**	F	66	74	162	7	L	Dysvascular disease	Aesthetic	Walker
**S12**	M	67	65	172	6	L	Dysvascular disease	Aesthetic	Walker
**S13**	M	49	93	178	55	L	Traumatic	Mobile knee	-
Mean (SD)		61.46 (11.12)	79.62 (16.84)	170.92 (7.65)	18.46 (21.46)				

F: Female; L: Left; M: Male; R: Right; SD: Standard Deviation; TFA: Transfemoral Amputation; TTA: Transtibial Amputation.

Table 2: Sensory and motor thresholds of the intact and the amputated limbs of the enrolled participants

	**Sensory threshold [mA]**		**Motor threshold [mA]**	
**ID**	**Intact limb**	**Amputated limb**	**Intact limb**	**Amputated limb**
**Group 1: TTA**				
**S1**	2.00	2.50	9.00	12.00
**S2**	1.50	2.50	8.50	9.00
**S3**	2.50	2.50	11.00	12.00
**S4**	2.50	3.00	9.50	9.50
**S5**	1.50	3.00	9.00	11.00
**S6**	1.50	5.00	9.00	14.00
Mean (SD)	1.92 (0.49)	3.08 (0.97)	9.33 (0.88)	11.25 (0.84)
**Group 2: TFA**				
**S7**	4.00	9.00	14.00	15.00
**S8**	2.00	2.00	5.50	5.50
**S9**	2.50	3.00	7.50	11.50
**S10**	2.50	3.50	9.00	12.50
**S11**	3.00	3.00	8.50	12.50
**S12**	2.50	3.50	9.50	10.50
**S13**	1.50	2.00	3.00	4.00
Mean (SD)	2.57 (0.79)	3.57 (2.46)	8.14 (3.44)	10.21 (4.00)

SD: Standard Deviation; TFA: Transfemoral Amputation; TTA: Transtibial Amputation

Table 3: Characteristics of the elicited sensations during charge and frequency modulation of the tibial and sciatic nerve stimulation of the intact and the amputated limbs in the enrolled participants

**Charge Modulation**														
**Naturalness**					**Depth**					**Quality**				
	**TTA (66)**		**TFA (77)**			**TTA (66)**		**TFA (77)**			**TTA (66)**		**TFA (77)**	
	**Intact limb**	**Amputated limb**	**Intact limb**	**Amputated limb**		**Intact limb**	**Amputated limb**	**Intact limb**	**Amputated limb**		**Intact limb**	**Amputated limb**	**Intact limb**	**Amputated limb**
Natural	84%	82%	77%	72%	Superficial	91%	94%	83%	64%	Nothing	11%	11%	11%	11%
Almost natural	13%	9%	17%	28%	Deep	9%	6%	17%	17%	Pinch	14%	16%	15%	15%
Possibly natural	0%	0%	0%	0%	Both	0%	0%	0%	19%	Touch & Pression	0%	0%	3%	2%
Almost unnatural	3%	9%	6%	0%						Tingling	58%	17%	41%	30%
Unnatural	0%	0%	0%	0%						Vibration	17%	56%	30%	42%
**Frequency Modulation**														
**Naturalness**					**Depth**					**Quality**				
	**TTA (60)**		**TFA (70)**			**TTA (60)**		**TFA (70)**			**TTA (60)**		**TFA (70)**	
	**Intact limb**	**Amputated limb**	**Intact limb**	**Amputated limb**		**Intact limb**	**Amputated limb**	**Intact limb**	**Amputated limb**		**Intact limb**	**Amputated limb**	**Intact limb**	**Amputated limb**
Natural	83%	75%	78%	65%	Superficial	90%	100%	58%	65%	Nothing	0%	0%	0%	0%
Almost natural	10%	18%	17%	28%	Deep	10%	0%	36%	13%	Pinch	20%	0%	0%	0%
Possibly natural	0%	0%	0%	0%	Both	0%	0%	6%	22%	Touch & Pression	0%	25%	0%	0%
Almost unnatural	7%	7%	5%	7%						Tingling	30%	42%	67%	41%
Unnatural	0%	0%	0%	0%						Vibration	50%	33%	33%	59%

The total number of trials for the charge (Group 1: 66, Group 2: 77) and frequency (Group 1: 60, Group 2: 70) modulation in naturalness and depth differs from that of quality because the participants did not always feel a sensation in response to a stimulus (TFA: Transfemoral Amputation; TTA: Transtibial Amputation).
